# Effect of Recycling on the Mechanical Properties of 6000 Series Aluminum-Alloy Sheet

**DOI:** 10.3390/ma16206778

**Published:** 2023-10-20

**Authors:** Daniele De Caro, Michele Maria Tedesco, Jaume Pujante, Andrea Bongiovanni, Giovanni Sbrega, Marcello Baricco, Paola Rizzi

**Affiliations:** 1 Stellantis, Metals & Anticorrosion Department, Corso Settembrini 40, 10135 Turin, Italy; daniele.decaro@unito.it (D.D.C.); michelemaria.tedesco@crf.it (M.M.T.); 2 Eurecat, Centre Tecnològic de Catalunya, Unit of Metallic and Ceramic Materials, Plaça de la Ciència, 2, 08243 Manresa, Spain; jaume.pujante@eurecat.org; 3 Chemistry Department and NIS-INSTM, University of Turin, Via Pietro Giuria 7, 10125 Turin, Italy; andrea.bongiovanni@unito.it (A.B.); paola.rizzi@unito.it (P.R.); 4 Profilglass S.p.a, Via Meda, 28 (Zona Ind.le), 61030 Bellocchi di Fano, Italy; giovanni.sbrega@profilglass.it

**Keywords:** secondary aluminum, circular economy, formability, mechanical properties, 6000 series aluminum alloy

## Abstract

Sustainability is one of the biggest values of today and for the future of our society; a responsible usage of material in every sector is fundamental to achieving sustainability goals. Aluminum alloys are some of the most promising materials in terms of strength and weight, but their production implies the emission of a high amount of CO_2_. For that reason, the study and development of aluminum alloys with increasing scrap content play a central role in future applications. In the current study, two sheet-aluminum 6181 alloys with different scrap content were analyzed and compared with a 6181 alloy coming from primary production. The alloys were compared in terms of chemical composition, microstructure, tensile properties, and forming behaviors. The results showed that the alloys coming from secondary productions contained a higher amount of manganese, iron, and copper. The metallurgical and mechanical behaviors were very similar to those of the primary produced alloy. Nevertheless, a drop in formability was shown in the aluminum alloys containing a high scrap amount when stressed in a biaxial condition. The study demonstrated the viability of 6181 alloy production using a high scrap amount, highlighting the main difference with the same alloy coming from primary route production.

## 1. Introduction

One of the main drivers for the automotive industry nowadays is the weight reduction of every vehicle part to decrease the fuel consumption, and so reduce CO_2_ emissions of internal combustion engine vehicles, and, for full battery electric vehicles, increase energy efficiency and battery duration [1]. Connected to this target, the automobile sector is going, in the coming years, to become a carbon-free industry (by 2038 for Stellantis), and raw-materials decarbonization will play a key role in achieving this target. The use of aluminum alloys in the automotive sector, correlated to their average weight in the car, represents one of the main contributions to the reduction of CO_2_ emissions, being around 20% of total car emissions. In fact, transport represents the widest sector for the usage of aluminum alloys, with a share of 40% of the annual production [2].

The introduction of aluminum alloys in the automotive sector began in the 1970s, when, due to the oil-price crisis, manufacturers began to lighten cars to improve fuel efficiency. The first applications concerned radiators, cylinder heads, and crash beams. Over the years, the average aluminum content per vehicle has continued to grow, extending into more and more car components.

In terms of volumes, the main aluminum alloys used are in the form of casting, which represents around 65% of the weight of cars produced in Europe. In fact, the use of aluminum alloys in flat products (i.e., sheet metal) today constitutes 18 wt% of the average of European cars and is constantly growing, and they will represent one of the fundamental materials of electric cars [2]. The components of the vehicle in which an extensive application of aluminum sheets is expected are the so-called auto body panels (ABS), which represent all the moving components of the car (doors, bonnet, trunk, etc.).

Considering the entire market (including battery electrical vehicle, BEV), the average usage of aluminum alloys is currently (2021) 179.2 kg per vehicle, for an average total mass of 2989 kg [3]. It is expected that by 2025 there will be an increase of 20 kg of aluminum alloys per vehicle, considering the new applications for BEV, like the battery tray.

Lightweight aluminum sheets are used for vehicle applications due to several benefits: limited cost for weight reduction compared to steel, improvement of vehicle efficiency, and reduction of the total greenhouse gas emission throughout the life of the vehicle. Moreover, aluminum sheets can meet important requirements for the applications of car components, such as strength, formability, joining, painting, and adhesive suitability [4].

The currently most used aluminum alloys in the automotive sector are AlMgSi 6000 and AlMg 5000 series, in particular for body-in-white (BIW) applications [5]. The 6000 series are generally applied on outer parts for aesthetic reasons and 5000 series are used for inner components. They are processed through cold stamping technology to produce the components and are assembled using resistance spot welding and hemming processes.

The sustainability of aluminum alloys will be a hot topic in the coming decades. In fact, it will not be enough to lighten the vehicle, but it has to be obtained in a sustainable way, using materials with a low CO_2_ impact. Producing primary aluminum is expensive in terms of raw material (a process with a total efficiency of about 25%), electricity, and in terms of CO_2_ emissions. Unfortunately, in the aluminum industry, the predominant energy resource is coal, resulting in high emissions during production.

There are basically two strategies to make the production and use of aluminum alloys sustainable, both contributing to the same goal. The first strategy is to increase the use of renewable energy sources to produce primary aluminum. Unfortunately, this is not enough to achieve zero emissions. The second strategy is to transform the business model from linear to circular. In other words, to recycle as much as possible aluminum alloys used in cars, to be used for new components in new vehicles, without degrading the expected performances. The recycling of aluminum alloys includes the collection of intermediate waste or preconsumer scrap (stamping scraps) and end-of-life waste (aluminum alloys at the end of the product life). Producing new aluminum alloys from recycled material has a huge energy advantage. In fact, only 5% of the energy is required when compared to the primary aluminum alloy production [6,7].

On average, one-third of aluminum alloys used today are produced from recycled scraps [8]. However, this fraction is expected to increase up to 50% by 2050, according to the data (2019) from the International Aluminum Institute [6,9,10]. Due to the advantages, in terms of greenhouse gases and energy consumption, secondary aluminum alloys represent an important pillar of the circular economy and a driver for sustainable metallurgical research [11,12,13].

Scraps play a significant role in the global aluminum industry, as a valuable source of raw material. They are commonly used in recycling processes to produce new aluminum products, reducing the need for primary aluminum extraction and minimizing environmental impacts.

The current situation regarding aluminum scraps depends on several factors, including market demand, recycling rates, and global economic conditions. Increased awareness of sustainability and environmental concerns has led to a growing emphasis on recycling aluminum scraps.

However, certain challenges can impact the aluminum-scrap scenario in the future. These challenges include:

- Supply and Demand Imbalances. Fluctuations in the availability of aluminum scraps can affect price and demand. Factors such as economic downturns, changes in manufacturing practices, and global trade policies can disrupt supply chains and lead to imbalances;
- Quality Control. Ensuring the quality and purity of aluminum scraps is crucial for effective recycling. Contaminations with other materials or impurities can reduce the scraps’ value and can make them more challenging to process;
- Collection and Sorting Infrastructure. Efficient collection and sorting systems are necessary to maximize the recovery of aluminum scraps. Developing and maintaining proper infrastructures, such as recycling facilities and sorting technologies, can be a challenge in some regions;
- Technological Advancements. Innovations in recycling technologies can enhance the efficiency of aluminum-scrap processing. However, implementing these advancements on a large scale and ensuring their economic viability can pose significant challenges.
- Global Trade Dynamics. Changes in trade policies, tariffs, and regulations can impact the movement of aluminum scraps across international borders. Such factors can introduce uncertainties and can affect the pricing and availability of scraps.

In the recycling of scraps, it is necessary to differentiate between preconsumer scraps, produced during processing and often characterizing well-defined alloy classification, and low-quality mixed postconsumer scraps, which must be sorted before remelting. About the two different types of scrap mentioned above:

- Postconsumer scrap is a contaminated and mixed low-price scrap coming from end-of-life products;
- Preconsumer scrap is a well-defined high-quality secondary material coming from a closed loop with customers.

Today, no real closed loop is applied, and the aluminum scraps coming from one sector are generally degraded to produce aluminum alloys, with decreased performances suitable for a different sector. This problem becomes more complex for vehicles where multimaterials concepts are applied [14,15].

The main concern about using secondary aluminum for flat products in automotive applications is related to the amount of trace elements that can be tolerated in existing alloys without large performance deterioration. Impurity elements could primarily affect the alloy formability during stamping operation. Most of the attention is about the Iron content that tends to increase in recycled aluminum, manifesting as iron intermetallic particles dispersed in the aluminum matrix when the iron content is higher than 0.3 wt%. These intermetallic particles can affect both the local and global formability of the alloy, as demonstrated in the literature [4,5,6,7,8,9,10,11,12,13,14,15,16,17,18]. Moreover, the iron-based intermetallic phase could lead to intergranular fracture affecting the failure mechanism of the material.

Copper is another impurity element that is generally very limited in 6xxx wrought alloy (<0.1 wt%) due to possible corrosion-properties deterioration. Nevertheless, copper promotes strength in 6xxx alloys because it introduces the Q phase and its precursor [4].

Another element whose content could increase due to the recycling process is manganese, which could have a beneficial effect on the final alloy due to the modification in the shape of the plate-like iron particles in less damaging equiaxed morphology [4].

Considering the current scenario, three steps are necessary to increase flat aluminum-alloy sustainability:

1. Reduce the alloy’s family in the vehicle, i.e., ideally move the future production to the use of only the 6xxx alloy family;
2. Design more impurity-tolerant aluminum alloys, with performances comparable to those obtained in alloys obtained with primary route production;
3. Improve the technology for scrap sorting.

In this work, the mechanical and microstructural properties of 6181 T4 aluminum alloys with different content of scraps are investigated, and the results are compared with those obtained from a 6181 T4 aluminum alloy coming from primary production (i.e., scrap content below 20%) currently used in standard automotive production. The aim of the study is to prove that sheets of aluminum alloy 6181 produced with high recycling content can have similar performances with respect to 6181 produced using primary aluminum.

## 2. Materials and Methods

Two sheets of aluminum alloy 6181 were supplied by Profilglass, containing different levels of scrap content:

- 6181-A, with 70% of scrap content;
- 6181-B, with 85% of scrap content.

The used scrap composition is reported below:

- Preconsumer scrap, mix of 5xxx and 6xxx, 70%;
- Postconsumer scrap, coming from end-of-life products, 30%.

The postconsumer scraps came from Zorba scrap steam.

The two variants were compared with a 6181 alloy coming from a primary source, containing a scrap content lower than 20%, and today used in current automotive production (6181-R).

The wrought alloys were produced in the T4 state. A foundry recipe was defined with the mixture of scrap proposed by the Salema project. The casted slabs were solubilized before hot rolling at a temperature of 570 °C with a heating ramp for 150 °C/h for a soak time of 300 min and then hot rolled to an 8 mm thickness. The cold rolling reduced finally the thickness from 8.0 mm to 0.9 mm in 6 steps. To get the temper status T4, a solubilization heat treatment (SHT) was performed in a continuous floating furnace, with a soak temperature of 560 °C for 25 s with a cooling rate > 25 °C/s.

### 2.1. Mechanical Test

**Figure 1:**
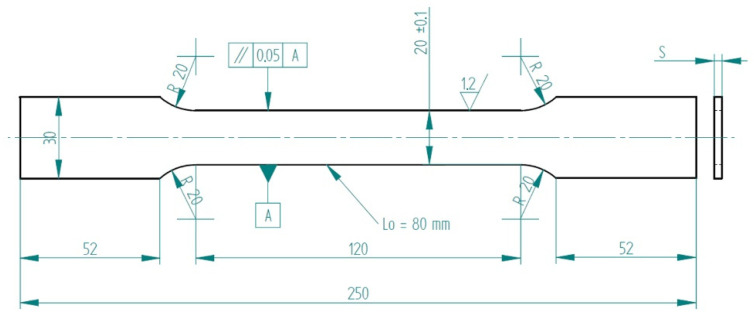
Tensile test specimen.

Samples for the tensile test were cut by using a milling operation in longitudinal, transverse, and diagonal directions with respect to the rolling direction. Three samples for each direction and alloy were produced. Specimens were produced according to Figure 1.

The tensile tests were performed by an electromechanical universal testing machine with a 200 kN maximum load capacity. A mechanical extensometer was used in the longitudinal and transversal directions to measure the strain. The speed of the cross head is 0.75 mm/min in the elastic region and 6 mm/min in the plastic region.

**Figure 2:**
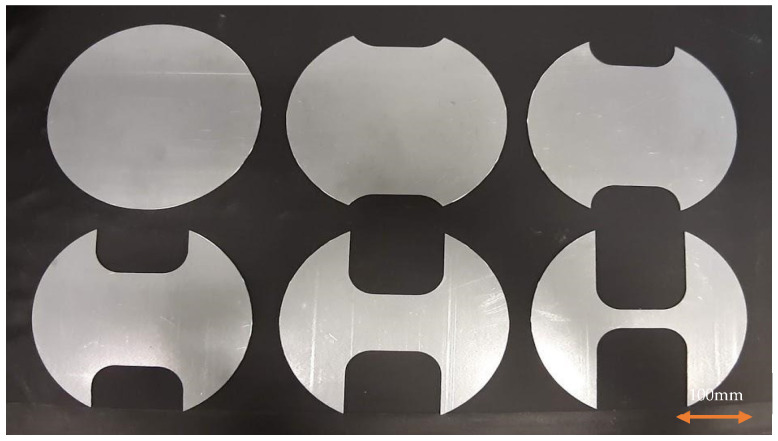
Nakazima test specimen.

To assess the failure criteria during forming operation, a Nakazima test, according to the standard ISO 12004-2 [19], was performed. The goal of the test is to determine the forming-limit curve of the material. The formability limit curve (FLC) is a curve that represents the maximum deformation that can be reached by the material without incurring fracture. In order to provide a more accurate definition, FLC can be defined as the locus of points in the plane below which the specimen deforms without undergoing necking or breaking, referring more correctly to primary deformation EPS1, in the direction of the largest diameter of the specimen used, and secondary deformation EPS2 in a direction perpendicular to the previous one. The resulting quantities are dimensionless. An example of a specimen for the FLC test is reported in Figure 2.

The test consists of deforming the specimens using a hemispherical punch and, at the same time, keeping the edges of the specimens blocked to prevent the sheet from sliding.

Samples for forming tests were cut in a transversal direction with respect to the rolling direction, which represents the preferred fracture direction for aluminum alloys. To perform the test, an Erichsen machine was used. The strain on the samples was analyzed through a 3D Digital Image Correlation device.

### 2.2. Optical Microscope and Fracture Surface Analysis

Metallographic analyses were performed with an optical microscope after Barker etching. The solution for etching is composed of 1.8% fluoroboric acid in water and applying a tension in a continuous current of 20–45 V dc, for up to 2 min.

All samples were tested in the T4 condition (solubilization, tempering, and natural aging).

The grain size has been determined according to ASTM (American Society for Testing and Materials International, West Conshohocken, PA, USA) size. Reported values correspond to the number of grains per square inch at 100X magnification.

The fracture surfaces were observed by means of a scanning electron microscope (SEM) (Zeiss Evo MA 25). Moreover, a microanalysis using energy-dispersive X-ray spectroscopy (Oxford AZtec Energy X Max 80) was carried out on the fracture surface to detect the elements’ distribution.

## 3. Results and Discussion

### 3.1. Chemical Analysis Results

The alloys were analyzed with optical emission spectroscopy (OES) to detect the chemical composition. The results are shown in Table 1.

The analysis shows that the 6181 alloys produced with higher scrap content present a high content of elements like iron, manganese, and copper, as expected. The copper and manganese content exceeds the maximum value allowed for standard 6181 reported in ISO 573-3 [20], while iron remains inside the standard composition.

Scraps used for the production of secondary alloys were extracted from the Zorba stream, using semiautomated methods.

Before using the scrap, a basic study was performed consisting of extracting ten fragments at random and analyzing their composition by means of OES (Table 2). All fragments could be identified as consisting of 5XXX or 6XXX alloy. Moreover, for the production of the final alloy, more copper has been added in order to take into account the future worst scenario in scrap quality.

### 3.2. Optical Microscope Analysis Results

**Figure 3:**
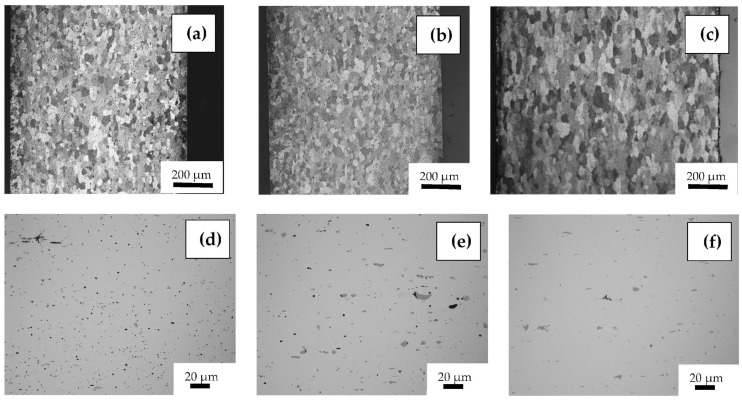
Metallographic cross sections of the studied materials: Top row, optical microscopy images of (**a**) 6181-A; (**b**) 6181-B; (**c**) 6181-R; Bottom row, optical microscope detail (**d**) 6181-A; (**e**) 6181-B; (**f**) 6181-R.

The optical microscopy metallographic analysis of 6181-A and 6181-B show a homogeneous grain distribution, with an ASTM size of nine (Figure 3a,b). Alloy 6181-R exhibits bigger grains with an ASTM size of eight.

**Figure 4:**
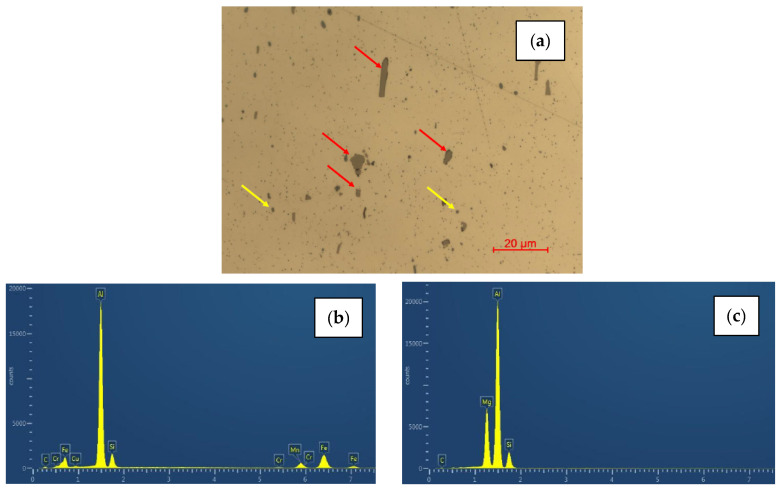
EDS analysis on second particles. (**a**) Second particles at higher magnification, red arrow larger particles, and yellow arrow small-rounded particles. (**b**) EDS analysis on large particles consisting basically of Al-Si-Fe-Mn compositions. (**c**) EDS analysis on small particles containing mainly Al, Si, and Mg.

A detailed optical microscope analysis was performed to verify the presence of phases caused by residual elements, most notably iron (Figure 3d–f). The alloys with higher recycled content 6181-A and B showed a higher presence of second particles. Moreover, these inclusions appear to be larger and more clustered than in 6181-R. These particles were analyzed by means of SEM/EDS (Figure 4). This semiquantitative analysis showed two different kinds of particles, consisting basically of Al-Si-Fe-Mn compositions (larger particles), more abundant in 6181-A and -B than in the reference 6181-R samples, and small rounded particles containing mainly Al, Si, and Mg.

### 3.3. Tensile Test Results

The results of the tensile tests obtained for the three 6181 alloys in different directions are reported in Table 3. The average value of three tested samples is reported, together with the error, showing a small scattering of results. Each alloy shows similar mechanical properties along the different testing directions, thus evidencing a nearly isotropic behavior. The observed results can be explained by considering the T4 treatment, which, through the solution heat treating temper condition, produces a complete recrystallization of the grains and produces a homogeneous microstructure in the sheet metal.

**Figure 5:**
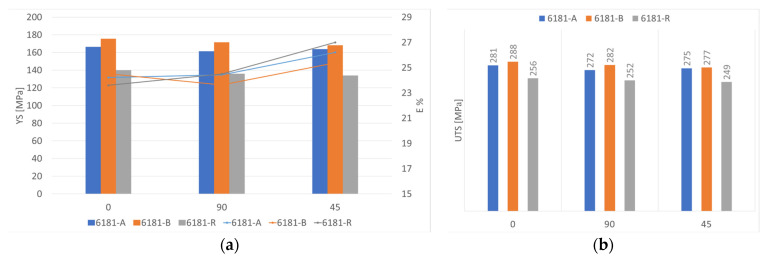
Comparison of mechanical properties for recycled and reference alloys: (**a**) YS and E% (**b**) UTS.

In comparing the three different samples, the results show that increasing the scrap content increases the material strength, about 20 MPa, with no negative effect on the ductility [16,17]. It must be noticed that the scrap-based alloys contain a higher amount of both magnesium and copper with respect to the primary alloy, both increasing strength in treatable aluminum alloys. In particular, copper content is higher than the accepted standard (see Table 1). A comparison of mechanical properties is reported in Figure 5.

The major effect of the instruction of scraps in the alloy is on the work-hardening exponent n. Work-hardening exponent n is defined according to Hollomon law:

(1) $$\sigma_{T}=K\epsilon^{n}$$

**Figure 6:**
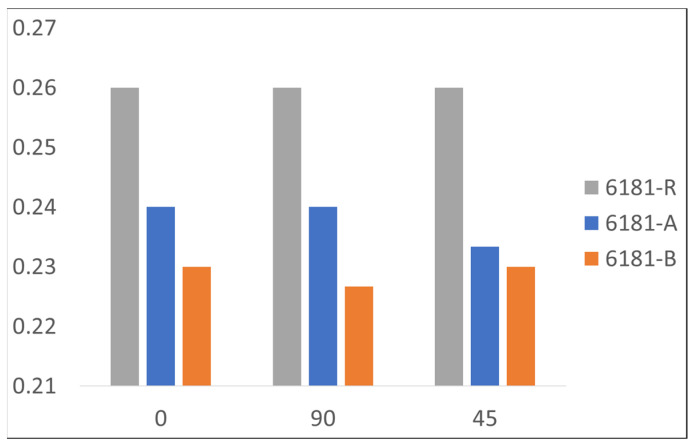
Hardening exponent “n” for each alloy at different testing directions.

Work hardening exponent n, as defined in Equation (1), has a big effect on flat metal stamping behavior [18]. The aluminum alloy, in the most deformed area, is strengthened by work hardening to reduce strain localization. By decreasing the work-hardening exponent, the most deformed zone will become thinner at high deformation rates, leading to the fracture of the component. Increasing the value of the work-hardening exponent, the material becomes stronger in the most deformed zone, reducing the tendency to localized thinning. A detailed trend for the hardening exponent is reported in Figure 6 in comparison to the scrap content of the alloy.

As shown, the n exponent decreases with increasing the scrap content, with a potential drawback for the formability of the alloy in the stamping operation.

**Figure 7:**
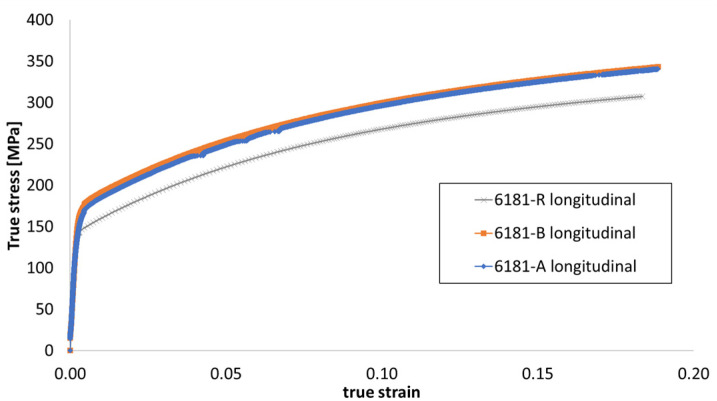
True stress–true strain curves for each alloy at a longitudinal testing direction.

A comparison of the true stress–strain curve for each alloy in a longitudinal direction is reported in Figure 7.

**Figure 8:**
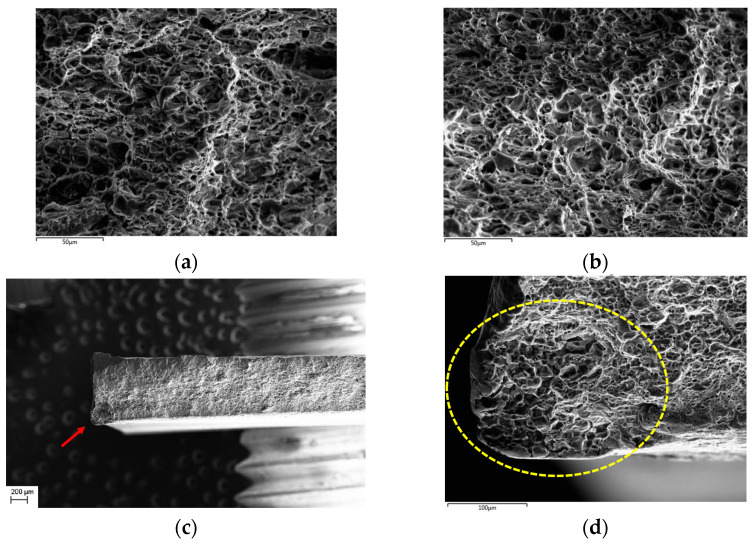
Fracture-surface analysis: (**a**) 6181-A; (**b**) 6181-R; (**c**) 6181-B low magnification, with red arrow pointing to intergranular fracture; (**d**) 6181-B high magnification with the yellow circle around the intergranular fracture zone.

The fracture surfaces for samples 6181-A, 6181-B, and 6181-R tested in a transversal direction were observed by means of field-emission scanning electron microscopy. The images (Figure 8) show that on 6181-A and 6181-R the fracture is entirely with a ductile morphology; otherwise, in 6181-B, with the highest scrap content (85%), there are some traces of intergranular fracture morphology [21].

The intergranular fracture zone is present in the 6181 B alloy near the edge of the tensile sample, as shown in Figure 8d.

A deeper analysis with EDS has been carried out to check for the presence of intermetallic phases in the intergranular fracture zone and to compare the element distribution among alloys 6181-A, 6181-B, and 6181-R.

**Figure 9:**
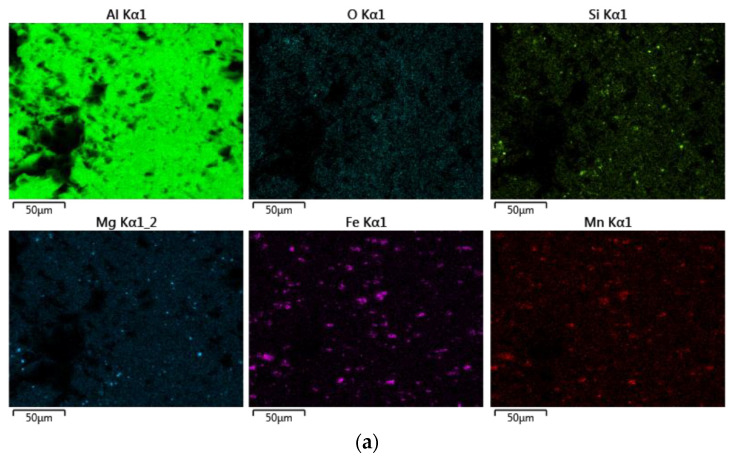

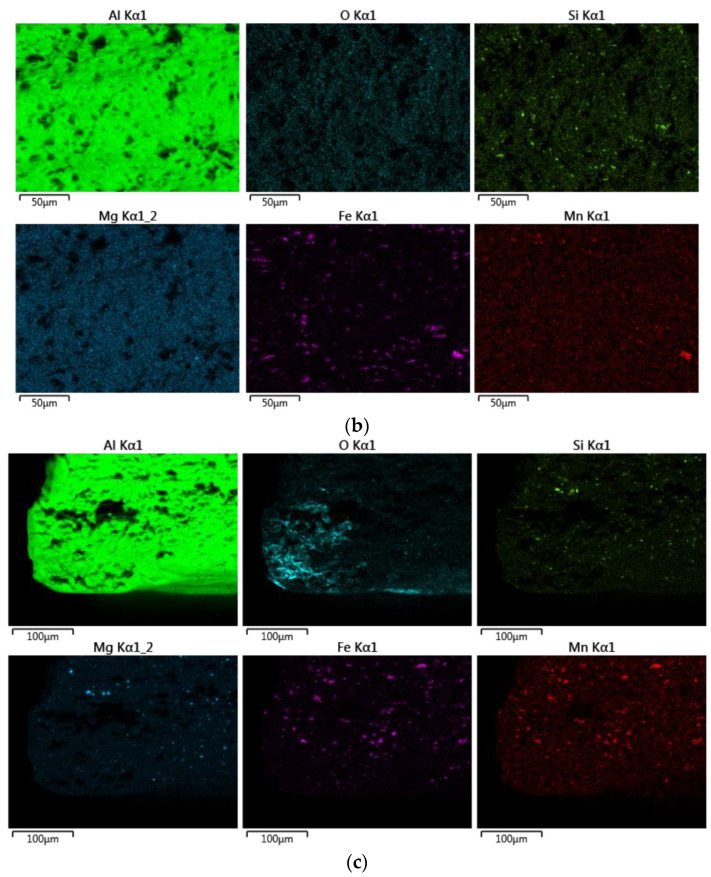
EDS analysis: (**a**) 6181-A; (**b**) 6181-R; (**c**) 6181-B.

The element distribution on the surface fracture is reported in Figure 9.

From the EDS analysis, it is evident that the highest oxygen concentration is present in the alloy 6181 B in the intergranular fracture zone, but, from the acquired data, it was not possible to determine the possible cause of this increased oxidation. Moreover, neither intermetallic particles nor anomalies in the elements’ distribution are detected in the intergranular fracture zone. Even if the alloy 6181-B, with higher scrap content and higher iron content (0.35 wt%) does not contain detectable intergranular phases, their presence in a low amount and with a small size can influence the mechanical behavior of the alloy, even if not detectable with EDS maps.

### 3.4. Forming-Test Results

**Figure 10:**
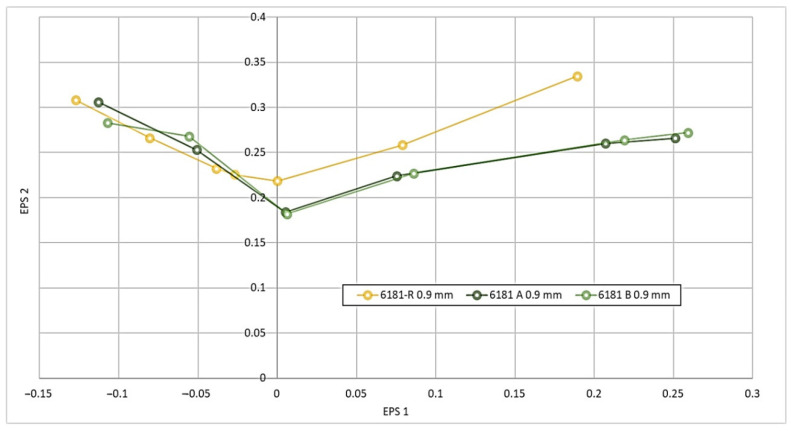
Forming-limit diagram.

The results of the Nakazima tests are reported in Figure 10, which show that the FLC of the three 6181 alloys is similar in the left part of the diagram, representing the uniaxial stress–strain state, as confirmed also by tensile test results. Otherwise, in a biaxial condition, the 6181-R alloy, with scrap lower than 20%, exhibited better forming behavior. The higher levels of iron in 6181-A and 6181-B lead to lower forming behaviors in a biaxial stress–strain condition, as reported in recent works [18,21].

Presented results show that the increase in scrap content leads to a higher amount of impurity elements (iron, manganese, and copper) in the alloy composition. The higher level of impurities does not have any negative effect on the microstructure of the alloys. Moreover, the higher scrap content leads to a beneficial effect in terms of the mechanical strength of the material, with no negative effect on elongation at the ruptures. With the highest amount of scraps (85%), the samples after tensile tests showed, in some zones, an intergranular fracture, which is not common for this alloy. The results of the Nakazima test of the recycled alloys were similar to those of the primary aluminum alloy in a uniaxial condition, but worse values have been observed in the biaxial condition, showing that some differences with respect to primary production could occur in forming operations.

## 4. Conclusions

In this work, the possibility of high-performance aluminum alloys being produced using high amounts of scraps has been discussed. The main conclusions obtained are the following:

- Using a mixture of preconsumer scraps and postconsumer scraps extracted from the Zorba stream, it has been possible to produce a high-performance 6XXX-series aluminum alloy including scrap rates of up to 85%;
- The composition of alloy 6181 is quite tolerant to residual elements. However, even in this case and using well-selected scraps, some alloying elements are typically kept low, most notably Cu and Fe, which have increased beyond the levels typically used in the industry. Magnesium levels were also higher than expected, in this case, due to the high content of 5XXX series scraps in the mix;
- The high amount of scrap has apparently not affected the microstructure of the materials.
- Tensile properties did not significantly change, but a small increase in strength has been observed, probably linked to the increased Cu and Mg content. However, while ductility did not significantly change, defects in the form of inclusions could be found in the fracture initiations;
- Formability, as measured in the Nakazima test, was affected mainly in the biaxial range. Indeed, alloys including high scrap quantity performed worse than the reference. This change did not occur on the uniaxial range of the FLD curve. Nevertheless, formability may be sufficient for applications with low geometrical complexity;
- Overall, intensive use of recycling in cold-formed sheet aluminum can be realistically used as far as components to be produced have only moderate geometrical complexity. This opens the possibility of highly circular aluminum sheets entering mainstream use in passenger cars.

## Figures and Tables

**Table 1:** Alloy chemical composition wt%.

	Si	Fe	Cu	Mn	Mg	Cr	Zn	Ti	Al
6181-A	1.00	0.30	0.19	0.28	0.80	0.02	0.07	0.03	rest
6181-B	1.10	0.35	0.19	0.30	0.84	0.02	0.08	0.03	rest
6181-R	0.89	0.21	0.09	0.01	0.69	0.01	0.02	0.02	rest
6181 ISO 573-3	0.80–1.20	0.45 max	0.1 max	0.15 max	0.6–1	0.1 max	0.2 max	0.1 max	rest

**Table 2:** Chemical composition corresponding to a sampling of scrap fragments wt%.

Fragment	Si	Fe	Cu	Mn	Mg	Cr	Zn	Al
1	0.31	0.39	0.06	0.23	2.9	0.03	<0.03	rest
2	0.88	0.27	0.09	0.16	0.4	0.05	<0.03	rest
3	0.23	0.31	0.03	0.19	1.96	0.08	<0.03	rest
4	0.88	0.24	0.1	0.16	0.42	0.04	<0.03	rest
5	0.12	0.24	0.08	0.38	4.45	<0.03	<0.03	rest
6	0.31	0.41	0.05	0.28	3.00	0.03	<0.03	rest
7	0.98	0.26	0.09	0.16	0.36	0.04	<0.03	rest
8	0.22	0.31	0.03	0.25	1.94	0.08	<0.03	rest
9	0.09	0.3	0.03	0.33	3.9	<0.03	<0.03	rest
10	0.07	0.25	0.04	0.32	4.18	<0.03	<0.03	rest

**Table 3:** Mechanical properties in T4 temper condition after tensile test in different directions.

Alloy	Direction	UTS [MPa]	YS [MPa]	E%	r@10%	rm	n@4-20%
6181-A T4	0	281 ± 1	166 ± 1	24.2 ± 0.1	0.64	0.53	0.24
	90	272 ± 3	161 ± 2	24.4 ± 0.3	0.54		0.24
	45	275 ± 1	164 ± 2	26.2 ± 0.3	0.46		0.23
6181-B T4	0	288 ± 5	176 ± 5	24.5 ± 0.5	0.69	0.56	0.23
	90	282 ± 5	172 ± 6	23.6 ± 2.0	0.57		0.23
	45	277 ± 1	168 ± 2	25.4 ± 0.5	0.49		0.23
6181-R T4	0	256 ± 1	140 ± 2	23.6 ± 0.1	0.65	0.56	0.26
	90	252 ± 3	136 ± 1	24.5 ± 0.1	0.8		0.26
	45	249 ± 2	134 ± 1	27.0 ± 0.2	0.4		0.26

UTS, Ultimate tensile strength; YS, Yield Strength; E%, elongation at fracture; r@10%, anisotropy coefficient measured at 10% elongation; rm, normal anisotropy; n@4-20%, hardening exponent measured between 4% and 20% elongation.

## Data Availability

The data could be found in the SALEMA project website.
